# The effect of bone morphogenetic protein-2 on osteosarcoma metastasis

**DOI:** 10.1371/journal.pone.0173322

**Published:** 2017-03-06

**Authors:** Jonathan Gill, Patrick Connolly, Michael Roth, So Hak Chung, Wendong Zhang, Sajida Piperdi, Bang Hoang, Rui Yang, Hillary Guzik, Jonathan Morris, Richard Gorlick, David S. Geller

**Affiliations:** 1 Division of Pediatric Hematology/Oncology and Blood & Marrow Cell Transplantation, Children’s Hospital at Montefiore, Albert Einstein College of Medicine, Bronx New York, United States of America; 2 Kosin University College of Medicine, Busan, Korea; 3 Department of Orthopaedic Surgery, Montefiore Medical Center, Albert Einstein College of Medicine, Bronx, New York, United States of America; 4 Analytic Imaging Facility, Albert Einstein College of Medicine, Bronx, New York, United States of America; 5 Department of Molecular Pharmacology, Albert Einstein College of Medicine, Bronx, New York, United States of America; University of South Alabama Mitchell Cancer Institute, UNITED STATES

## Abstract

**Purpose:**

Bone Morphogenetic Protein-2 (BMP-2) may offer the potential to enhance allograft-host osseous union in limb-salvage surgery following osteosarcoma resection. However, there is concern regarding the effect of locally applied BMP-2 on tumor recurrence and metastasis. The purpose of this project was to evaluate the effect of exogenous BMP-2 on osteosarcoma migration and invasion across a panel of tumor cell lines *in vitro* and to characterize the effect of BMP-2 on pulmonary osteosarcoma metastasis within a xenograft model.

**Experimental design:**

The effect of BMP-2 on *in vitro* tumor growth and development was assessed across multiple standard and patient-derived xenograft osteosarcoma cell lines. Tumor migration capacity, invasion, and cell proliferation were characterized. In addition, the effect on metastasis was measured using a xenograft model following tail-vein injection. The effect of exogenous BMP-2 on the development of metastases was measured following both single and multiple BMP-2 administrations.

**Results:**

There was no significant difference in migration capacity, invasion, or cell proliferation between the BMP-2 treated and the untreated osteosarcoma cell lines. There was no significant difference in pulmonary metastases between either the single-dose or multi-dose BMP-2 treated animals and the untreated control animals.

**Conclusions:**

In the model systems tested, the addition of BMP-2 does not increase osteosarcoma proliferation, migration, invasion, or metastasis to the lungs.

## Introduction

Osteosarcoma is a rare primary bone tumor, making up less than 1% of cancers in the United States.[1] However, it is the most common primary bone malignancy in children and young adults, making up 3.4% of all childhood cancers and 56% of bone malignancies in children.[2] Chemotherapy has been shown to improve survival in patients with osteosarcomas, with surgical removal of the tumor being an essential component of treatment. [3] The survival for patients with osteosarcoma has remained unimproved for over 3 decades at approximately 60%. In patients who recur, 85% develop pulmonary metastases. [4]

Historically, extremity osteosarcomas were typically removed by performing amputations or disarticulations. [5] In the modern era, limb-salvage surgery offers patients an alternative to ablative procedures and is utilized in 90% of patients with extremity osteosarcomas. [6] These patients realize survival outcomes comparable to those who undergo amputations. Limb-salvage surgery is characterized by extirpation of the primary tumor and subsequent reconstruction, using either a structural allograft bone or an endoprosthesis. Allograft use offers certain benefits, including restoration of bone stock and the availability of soft-tissue attachments. Unfortunately, allograft reconstruction is subject to several well-described complications including infection, allograft fracture, and non-union. Non-union at the allograft-host bone junction has been seen in over 25% of limb-salvage patients undergoing chemotherapy. [7] Patients who experience non-union frequently experience pain, delayed return to activities of daily living and ultimately, additional surgery is required, leading to additional cost and burden for patients. [8]

Bone Morphogenetic Protein-2 (BMP-2) has been shown to stimulate bone growth. The Food and Drug Administration (FDA) has approved BMP-2 to be used in spinal fusion surgery, fixation of open tibial fractures, oral and maxillofacial surgery, and in the management of recalcitrant non-unions. [9] However, use of BMP-2 in the context of osteosarcoma remains extremely controversial. In large part, this is owing to the expression of BMPs and BMP receptors in osteosarcoma and the concern that exogenous BMP-2 may stimulate growth and proliferation of residual microscopic disease. [10, 11] The FDA has issued a “Black Box” warning indicating that BMP-2 should not be utilized in either patients who have a tumor within the area of implantation or in patients who have had a tumor removed the site of BMP-2 implantation. Nevertheless, this concern remains highly disputed, and there is evidence that BMP-2 may serve as driver of tumor cell differentiation rather than proliferation. We have previously demonstrated that exogenous BMP-2 administration did not increase local tumor recurrence rates with a xenograft murine model. [12] The effect of local exogenous BMP-2 administration on pulmonary metastases has not been previously described.

## Materials and methods

### Cell lines

Standard osteosarcoma cell lines, 143b, HOS, U2OS, Saos-2, and MG63 (ATCC), the metastatic cell line, SaOS-LM7 (provided by Dr. Eugenie Kleinerman, M.D. Anderson Cancer Center), and xenograft cell lines, OS17 and OS31, [13] were grown in Eagle’s Minimum Essential Medium (EMEM) and supplemented with 10% fetal bovine serum (FBS) and a combination of 100 U penicillin with 0.1mg/ml streptomycin (P/S). [14, 15] Cells were grown in a humidified condition of 95% air and 5% CO2 at 37°C. Once confluent, cells were washed with phosphate buffer saline twice, then trypsinized and resuspended in media. Metabolic activity rates for cell lines, with and without adding BMP-2, (BioPharma, Inc., Seoul, Republic of Korea) were measured every 24 hours by using MTT cell proliferation assay kit (ATCC, Manassas, VA) according to manufacturer’s instructions. Proliferation was measured by performing manual cell counts of cultures containing 1 × 104 cells/mL in 6-well plates. The same sets of cells mentioned above were used, with and without the addition of 2ug/ml of BMP-2. Viable cells were counted using trypan blue exclusion.

### In vitro assays

#### Gene expression quantification

To further characterize the osteosarcoma lines utilized, we quantified gene expression for BAMBI, SOST, and NOG across 5 standard and 3 xenograft cell lines using quantitative real-time PCR. The experiment was performed both with and without the addition of BMP-2 over a 48 hour period. RNA was extracted using PureLink RNA Mini Kit (Life Technologies, Grand Island, NY) and subsequently converted to cDNA using SuperScript III First- Strand Synthesis System (Life Technologies) according to the manufacturers’ instructions. Gene expression quantitation was carried out using a 7,500 Fast Real-Time PCR system and Taqman Gene Expression assay mix (Life Technologies, Grand Island, NY; Assay IDs: Hs03044164_m1 for BAMBI, Hs00228830_m1 for SOST and Hs00271352_s1 for NOG). The housekeeping gene GAPDH was used as a control and multiple wells of scrambled control were included as negative controls. Reactions for each sample were performed in triplicate. mRNA levels were quantified using ΔΔCt method as per the manufacturer’s instructions, 7500 Sequence Detection System (Life Technologies). MSCs were used as a calibrator.

#### Wound-healing assay

Random migration motility was measured via a wound-healing assay as described previously. [16] Cells were cultured in serum-free media overnight before creating wounds. Photos were taken at 0, 6, and 24 hours at the same region. The width of the scratch wounds was recorded with a Nikon TE200 inverted light microscope attached to a CCD (Diagnostic Instruments, Sterling Heights, MI).

#### 3-D spheroid assay

3D spheroid cell invasion assay was performed using Cultrex® 96-well 3D Spheroid BME Cell Invasion Assay Kit (Trevigen, Inc, Gaithersburg, MD) according to manufacturer’s instruction. Briefly, osteosarcoma cell lines in spheroid formation ECM were seeded 3000 cells per well in 3D culture qualified 96-well spheroid formation plate, followed by incubation at 37oC for 72 hours to promote spheroid formation. Upon completion of spheroid formation, the spheroid is embedded in an invasion matrix composed of basement membrane proteins. After allowing the matrix to form hydrogel network on which invasive cells can travel, 100 μl of cell culture medium containing chemoattractant and BMP-2 were added. Then each day until day 6 the morphology of spheroid of each well was photographed using the 4x objective of a Nikon Inverted Microscope ECLIPSE TE200 attached to a CCD camera (Diagnostic Instruments, Sterling Heights, MI). All cell lines were assayed in triplicate and qualitative analysis of the figures obtained was performed. Non-invasive cell line, MCF-7, and invasive cell line, MDA-MB-231, were included as a measure of quality assurance, and wells without the invasion matrix were also included as negative controls.

#### Boyden chamber

Haptotaxis, defined as cell movement towards an immobilized ECM protein gradient, was measured in the Boyden chamber system and was performed using the Chemicon QCM Quantitative Cell Migration Assay (Millipore, Billerica, MA). Cells were serum-starved overnight before being seeded into Boyden chambers. Cells that migrated outside the chamber were stained and extracted in 300 μL of extraction buffer. Absorbance at 562 nm was measured using a microplate reader (Bio-Rad, Hercules, CA), which indicates the relative number of cells that migrate out of the upper chamber and into the lower chamber, which contains the chemoattractant. The fibrosarcoma cell line, HT1080, was used as a positive control and the non-invasive cell line 3T3 was used as a negative control.

#### Matrigel invasion

*In vitro* invasion through matrigel was quantitatively measured using the Biocoat Matrigel Invasion Kit (BD Bioscience, San Jose, CA). Serum-starved cells were plated in the invasion chambers with an 8μm pore size polycarbonate membrane, over which a thin layer of matrigel matrix was applied. Invading cells migrate through the matrix layer and become attached to the bottom of the polycarbonate membrane, which is then stained, extracted, and measured using a microplate reader with an absorbance of 562 nm (Bio-Rad, Hercules, CA).

### Animal model

Experiments were performed with the approval of the Albert Einstein College of Medicine Institutional Animal Care and Use Committee (IACUC) and in accordance with the institutional animal welfare policy. Six to eight week-old female CB17 SCID mice were obtained (Taconic Farms, Germantown, NY) and housed in a pathogen-free barrier facility at all times. Heterotopic tumor implantation using SaOS-LM7 was effected via tail vein injection using a 26-guage needle. After standard alcohol preparation, approximately 10^6^ cells in 200μL of media were implanted using a 26-gauge needle. Tumor cells were allowed to grow for one week. Thereafter, 20 animals were randomized to either a treatment group (n = 10) or a control group (n = 10). The treatment group received a single 30μg tail vein injection of E. coli-derived BMP-2 (Kindly gifted by BioPharma, Inc., Seoul, Republic of Korea) that had been reconstituted in accordance with manufacturer’s recommendations to a concentration of 1mg/ml. The control group received no additional injections. Thereafter, animals were carefully observed for seven weeks, at which time they were euthanized by asphyxiation with 100% CO_2_. Body weight and harvested lung weight was recorded for each animal. The lungs were stained in Bouin’s solution and photographed. The experiment was repeated using three sequential 30μg BMP-2 tail vein injections, spaced in one-week intervals and compared with a control group that received no treatment.

### Animal care

Basic animal housing and maintenance was managed within the barrier facility by staff members of the animal institute in accordance with all rules and regulation of the facility. Animals were housed in cages with 5 animals per cage. The cages containing tumor bearing animals were clearly marked. All of the mice were observed daily including weekends by the PI’s staff. Body weight was checked twice weekly or as clinically indicated. Water was provided at all times. Feeding was in keeping with routine cycles and timing. A 12 hour light/12 hour dark cycle was utilized. PI’s staff did not enter the mouse room during the dark cycle. Temperatures of 65–75°F with 40–60% humidity are maintained. A warming lamp was available while the mice were anesthetized and through the recovery period. Upon recovery, mice were returned to their cages. Minimal blood loss was expected and anesthesia time was less than 1 minute. Animals were monitored for one hour after anesthesia to confirm normal behavior, then as regularly scheduled. Animals were routinely evaluated daily by both the investigators and the housekeeping staff. Any identification of an unhealthy or ill animal was immediately conveyed to the PI. Mice were assessed daily for ulceration and animal activity and were assigned a body conditioning score daily as well. Animals which became moribund, defined as weight loss exceeding 20% of starting weight and/or a significant decrease in activity, which developed dyspnea or which were assigned a body conditioning score of 2 were euthanized by asphyxiation with CO_2_. Two animals met the criteria for euthanasia prior to the study endpoint and were sacrificed.

### Statistical methodology

In vitro proliferation assays, migration assays, and invasion assays were all performed in triplicate for each dose level of BMP. The data was summarized by calculating means and standard deviations for each BMP dose level. Student t-test was utilized assessing the effect of each dose level of BMP on proliferation, migration, and invasion in treated cells compared with untreated cells. P value ≤0.05 was considered statistically significant. In vivo assays assessing the effect of single-dose and multiple-dose BMP on the development of lung metastases was determined utilizing 10 mice in each experimental arm and 10 mice in each control arm. Mean lung weight, mean body weight, and mean lung weight/body weight, as well as standard deviations, were calculated for each experimental and control arm. Percentage was defined as mean lung weight divided by mean body weight. Student t-test was utilized assessing the effect of single-dose and multiple-dose BMP on the development of metastases comparing lung weight and lung weight/body weight in mice in the experimental arms versus mice in the untreated arms. P value ≤0.05 was considered statistically significant.

## Results

### BMP-2 does not increase osteosarcoma tumorigenesis *in vitro*

#### BMP-2 does not increase osteosarcoma proliferation *in vitro*

**Fig 1:**
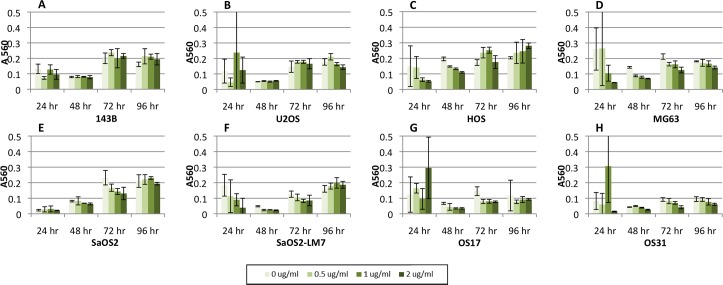
Effects of BMP-2 on the proliferation rate of different osteosarcoma cell lines. Four different amounts of BMP-2 were added and the proliferation rate was measured at 24, 48, 72, and 96 hours. (A) 143B (B) U2OS (C) HOS (D) MG63 (E) SaOS-2 (F) LM7 (G) OS17 (H) OS31. There were no changes in proliferation rates with increasing amounts of BMP-2.

Results following the addition of BMP-2 to eight cell lines (143b, HOS, U2OS, Saos-2, MG63, SaOS-LM7, OS17 and OS31) are summarized in Fig 1. Generally, as incubation time increased, the absorbance which serves as a measure of cell number, increased as well. Increasing doses of BMP-2 did not impact the metabolic activity or cell proliferation rates (S1 and S2 Figs). Proliferation and metabolic activity was measured after determining the seven of the eight cell lines Gene expression for BAMBI, SOST and NOG were increased following exposure to BMP2 across all tested cells lines with the exception of MG63(S3–S5 Figs).

#### BMP-2 does not increase osteosarcoma migration rate *in vitro*

**Fig 2:**
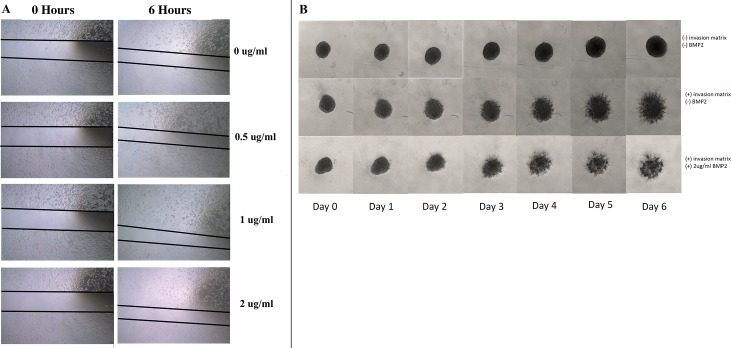
Wound-healing assay. 2A. A representative picture of wound-healing assay (HOS cell line) at 0 hour and 6 hour. This was used to qualitatively analyze effects of different concentrations of BMP-2 on motility (random migration). 2B. A representative picture of three-dimensional spheroid BME cell invasion assay (HOS cell line) with and without the addition of BMP-2. Images obtained on days 0 through 6. No qualitative differences in invasion were demonstrated.

**Fig 3:**
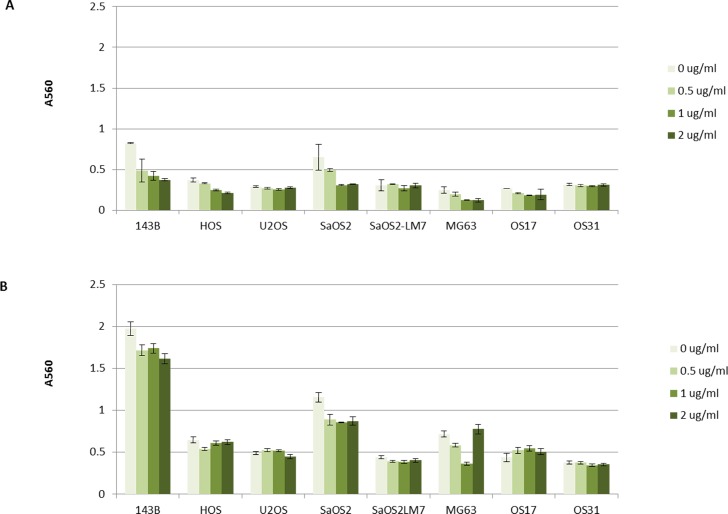
Effects of BMP-2 on migration capacity of osteosarcoma cell lines using a Boyden Chamber and chemotaxis cell migration assay. (A) at 5 hr, and (B) at 24 hour. Cells that migrated outside of the chamber were stained and extracted and cell migration was analyzed quantitatively by measuring absorbance at 560 nm.

Random cell migration was assessed using a wound-healing assay. The addition of BMP-2 (0.5, 1, and 2 ug/ml) did not yield a decrease in the scratch wound width across all 8 osteosarcoma cell lines (Fig 2A). Chemotaxis cell migration was assessed using a Boyden Chamber. The migration rate of osteosarcoma toward the chemoattractant did not increase with the addition of BMP-2 (0.5, 1, and 2 ug/ml) (Fig 3). Three-dimensional spheroid BME cell invasion assay with and without the addition of BMP2 demonstrated no qualitative differences in invasion across all tested tumor lines (Fig 2B and S6–S12 Figs).

The addition of BMP-2 to osteosarcoma cell lines does not increase random cell migration or cell migration in response to a chemical stimulus *in vitro*, regardless of dose used.

#### BMP-2 does not increase osteosarcoma invasion rate *in vitro*

**Fig 4:**
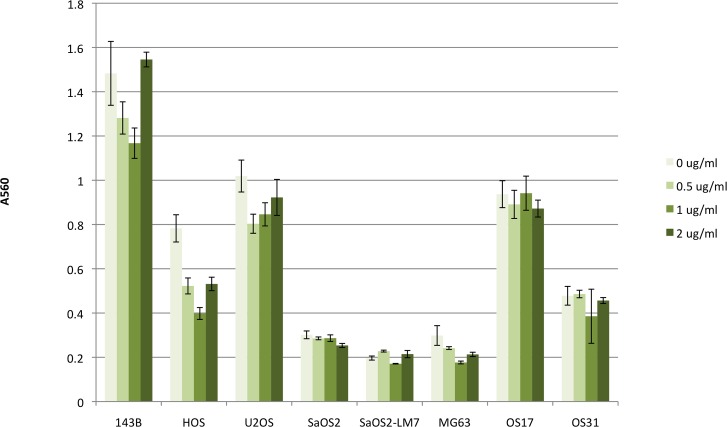
Effects of BMP-2 on cell invasion capacity of osteosarcoma cell lines. Cell invasion was determined using Biocoat Matrigel Invasion Kit and was measured quantitatively by absorbance at 560 nm. There was no significant increase in the number of invading cells with the addition of BMP-2.

Cell invasion was measured using a Biocoat Matrigel Invasion Chamber. Results of the experiment are summarized in Fig 4. There was no increase in the number of invading cells with the addition of BMP-2 at every dose tested, when compared to controls without the addition of BMP-2.

### BMP-2 does not increase osteosarcoma metastasis *in vivo*

#### Single-dose parenteral BMP-2 does not increase pulmonary tumor growth *in vivo*

**Fig 5:**
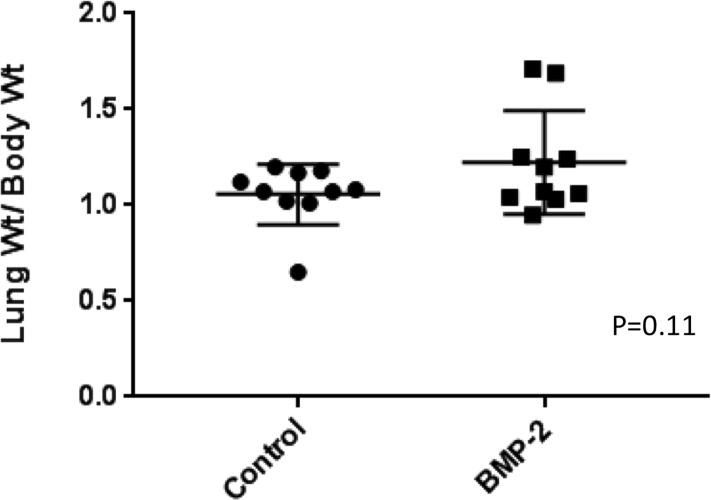
Single-dose BMP-2 treated animals bearing tumor (SaOS-LM7) compared to control animals. There was no significant difference in body weight, lung weight, or lung weight/body weight ratio between the two groups.

A xenograft murine model (SaOS-LM7) was used to assess osteosarcoma metastasis *in vivo* by measuring pulmonary tumor growth in mice. Animals treated with a single dose of parenterally administered BMP-2 were compared to animals which were untreated. The lung to body weight ratio was used to account for variances in weights between animals. Results are summarized in Fig 5. At 7 weeks post-implantation, there was no statistically significant difference in body weight (p = 0.8), lung weight (p = 0.09), or lung to body weight ratio (p = 0.11) between single-dose BMP-2 treated mice and control mice.

#### Multi-dose parenteral BMP-2 does not increase pulmonary tumor growth *in vivo*

**Fig 6:**
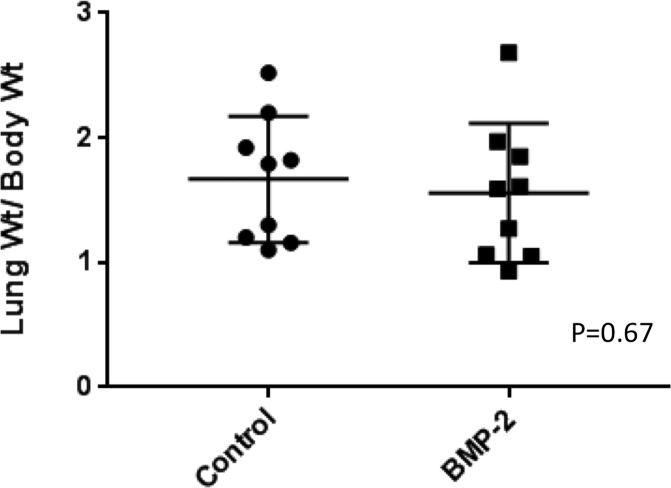
Multi-dose BMP-2 treated animals bearing tumor (SaOS-LM7) compared to control animals. There was no significant difference in body weight, lung weight, or lung weight/body weight ratio between the two groups.

A xenograft murine model (SaOS-LM7) was similarly used to assess osteosarcoma metastasis *in vivo* following triple-dose BMP-2 administration, given once per week. These animals were compared to animals which were untreated. Results of the experiment are summarized in Fig 6. Eight weeks post-tail-vein injection of osteosarcoma, there were no significant differences in body weight (p = 0.17), lung weight (p = 1), or in the lung to body weight ratio (0.67) between multi-dose BMP-2 treated mice and control mice.

## Discussion

Overall results from the *in vivo* and *in vitro* experiments in this study demonstrate that the addition of exogenous BMP-2 does not increase osteosarcoma proliferation, migration, invasion, or metastasis to the lungs. This finding is consistent with our previously reported results, which demonstrated that the local addition of exogenous BMP-2 failed to increase local recurrence rates. [12]

BMPs belong to the TGF-β superfamily and are involved in a broad array of cellular roles including development, proliferation, and differentiation. Both BMP-2 and BMP-7 demonstrate potent osteogenetic properties, and have been approved by the FDA for use in a number of clinical scenarios to promote bone healing. In theory, the use of these proteins at the time of surgical reconstruction could decrease the non-union rate at the allograft-host bone junction. However, as previously mentioned, the medical community remains exceedingly cautious about using BMP-2 in the context of osteosarcoma. BMPs and BMP receptors are expressed in osteosarcoma, which has lead to the concern that administering exogenous BMP-2 may promote tumor recurrence. [10, 11] In recent years, a number of reports have examined the role of BMP-2 in osteosarcoma. Several reports have linked a faulty BMP-driven differentiation mechanism to the development of osteosarcoma. For instance, Haydon and colleagues proposed that BMP-2, among other BMPs, drives osteogenic differentiation under normal conditions. They suggest that this effect is lost in osteosarcoma. [17] Luo and colleagues similarly implicate BMP-2 as a driver of tumor growth in osteosarcomas that harbor an error in terminal differentiation. [18]

Conversely, other investigators have characterized BMP-2 as an inhibitor of tumorgenesis in osteosarcoma. Wang and colleagues reported that BMP-2 treated severe combined immunodeficiency (SCID) mice did not develop tumors following heterotopic tumor implantation. They concluded that addition of BMP-2 results in inhibition of tumor-inducing gene expression and an upregulation of osteogenic differentiation markers. [19] Geng and colleagues showed that Coleusin factor inhibits osteosarcoma proliferation by upregulating BMP-2, leading to osteoblastic differentiation. In addition, the researchers were able to reverse this effect with the addition of a BMP-2 inhibitor, noggin. [20] Other investigators have found that BMP reduces tumor growth and increases the tendency for osteosarcoma to undergo apoptosis. [21] These studies suggest that normal BMP-2 acts as an osteosarcoma inhibitor and may drive differentiation, apoptosis, or both.

There are a number limitations of this study. The use of an immunocompromised xenograft model does not account for any potential confounding host factors associated with the administration of exogenous BMP-2. In addition, measuring lung metastasis by comparing the lung weight in BMP treated and control group mice is not the most sensitive method of analysis, and may not detect very small differences in the treated mice. During data analysis of the metabolic activity of the cell lines, there was a wide range of values. This may be due to cells remaining on the plate, a common effect demonstrated by solid tumors that prefer cell-cell interactions over cell-substratum interactions.

Previous experiments have demonstrated that the addition of exogenous BMP-2 does not increase the rate of local tumor recurrence. In this study, we likewise demonstrate that the systemic administration of exogenous BMP-2 does not increase metastasis in a xenograft model of osteosarcoma. We appreciate and agree with the concerns raised to date, which prioritize patient survival over functional outcome. That said, it is hoped that these results will drive future work intended to either demosntrate that BMP-2 is indeed cancer promoting or to demonstrate that BMP-2 can be safefly utilized within the context of osteosarcoma reconstructive surgery. Although we believe these results support the consideration of future clinical studies, we acknowledged that this is a very complex system and that the use of a single BMP is unlikely to fully quell the aformentioned concerns.

## Supporting information

S1 Fig Manual cell counts after exposure to BMP-2. For all four cell lines included the addition of BMP-2 did not results in increased proliferation of tumor cell lines.
(TIF)

S2 Fig Manual cell counts after exposure to BMP-2. For all four cell lines included the addition of BMP-2 did not results in increased proliferation of tumor cell lines.
(TIF)

S3 Fig Expression of BAMBI by the each cell line. There was a signficant difference in expression between the experimental group exposed to BMP-2 and the control group that was not exposed to BMP-2.
(TIF)

S4 Fig Expression of NOG by the each cell line. There was a signficant difference in expression between the experimental group exposed to BMP-2 and the control group that was not exposed to BMP-2.
(TIF)

S5 Fig Expression of SOST by the each cell line. There was a signficant difference in expression between the experimental group exposed to BMP-2 and the control group that was not exposed to BMP-2.
(TIF)

S6 Fig 3D assay of cell line 143B. The cell line without BMP-2 and invasion matrix, with invasion matrix and without BMP-2, and with BMP-2 and the invasion were compared and did not demonstrate a qualitative difference.
(TIF)

S7 Fig 3D assay of cell line U2OS. The cell line without BMP-2 and invasion matrix, with invasion matrix and without BMP-2, and with BMP-2 and the invasion were compared and did not demonstrate a qualitative difference.
(TIF)

S8 Fig 3D assay of cell line SaOS2. The cell line without BMP-2 and invasion matrix, with invasion matrix and without BMP-2, and with BMP-2 and the invasion were compared and did not demonstrate a qualitative difference.
(TIF)

S9 Fig 3D assay of cell line LM7. The cell line without BMP-2 and invasion matrix, with invasion matrix and without BMP-2, and with BMP-2 and the invasion were compared and did not demonstrate a qualitative difference.
(TIF)

S10 Fig 3D assay of cell line MG63. The cell line without BMP-2 and invasion matrix, with invasion matrix and without BMP-2, and with BMP-2 and the invasion were compared and did not demonstrate a qualitative difference.
(TIF)

S11 Fig 3D assay of cell line OS17. The cell line without BMP-2 and invasion matrix, with invasion matrix and without BMP-2, and with BMP-2 and the invasion were compared and did not demonstrate a qualitative difference.
(TIF)

S12 Fig 3D assay of cell line OS31. The cell line without BMP-2 and invasion matrix, with invasion matrix and without BMP-2, and with BMP-2 and the invasion were compared and did not demonstrate a qualitative difference.
(TIF)
