# Supplementary figures and images for: The effect of bone morphogenetic protein-2 on osteosarcoma metastasis
Source: PLoS One. 2017 Mar 6;12(3):e0173322. doi: 10.1371/journal.pone.0173322 (PMC5338793; doi:10.1371/journal.pone.0173322)

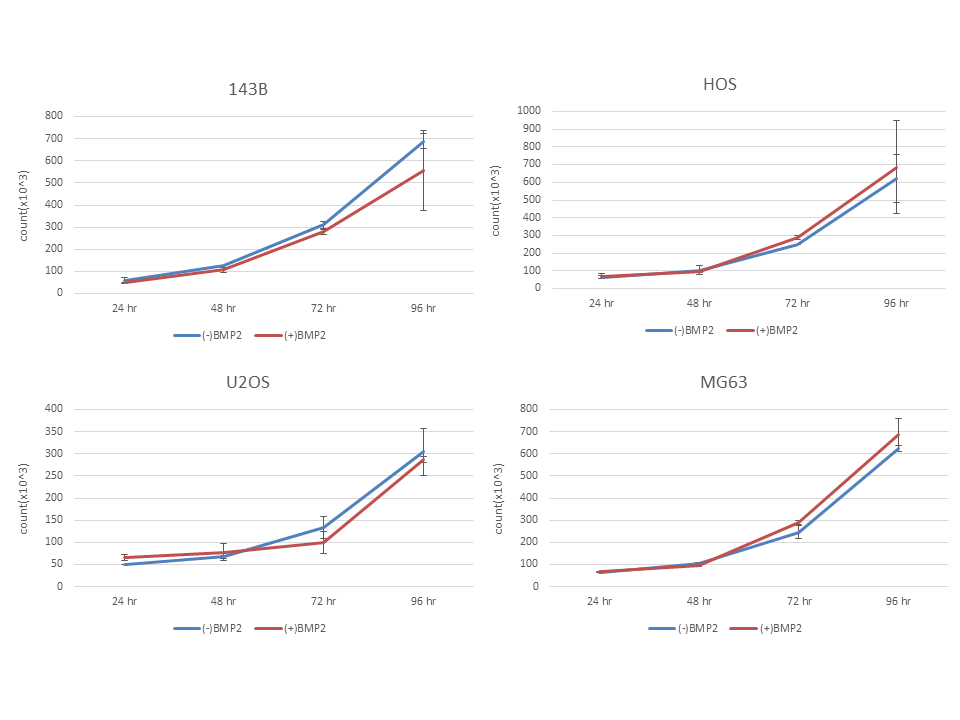

Supplement: S1 Fig — For all four cell lines included the addition of BMP-2 did not results in increased proliferation of tumor cell lines. (TIF) [file pone.0173322.s001.tif]

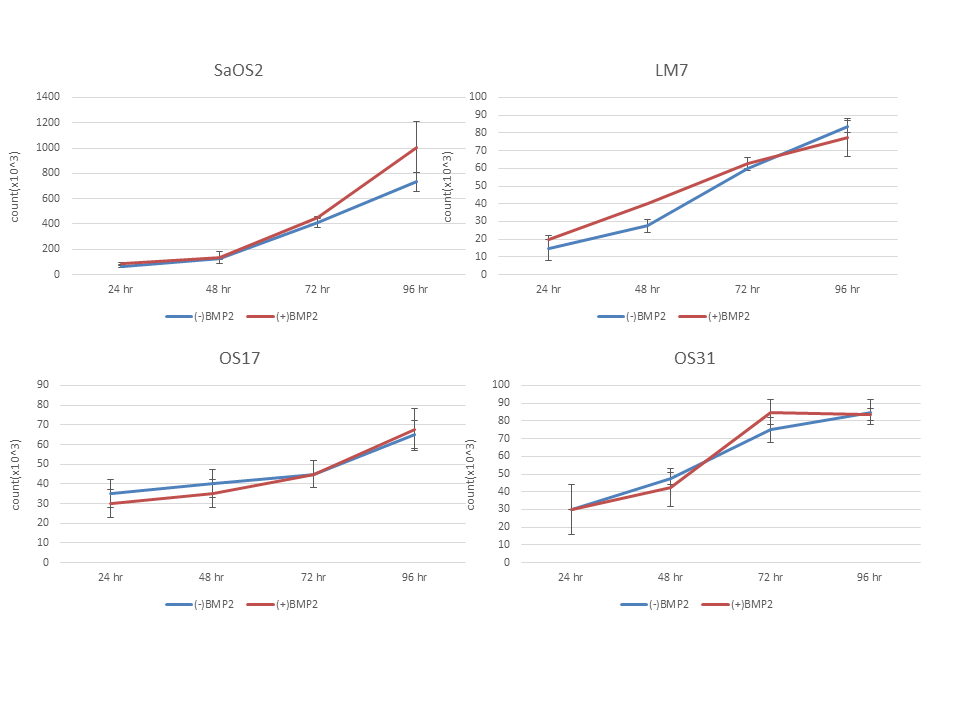

Supplement: S2 Fig — For all four cell lines included the addition of BMP-2 did not results in increased proliferation of tumor cell lines. (TIF) [file pone.0173322.s002.tif]

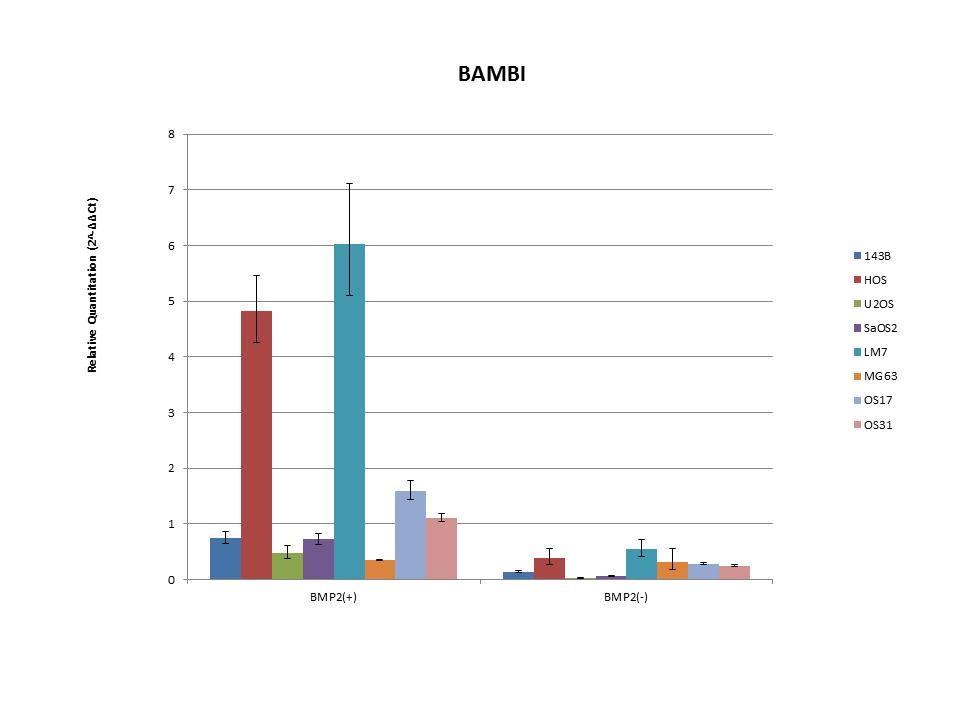

Supplement: S3 Fig — There was a signficant difference in expression between the experimental group exposed to BMP-2 and the control group that was not exposed to BMP-2. (TIF) [file pone.0173322.s003.tif]

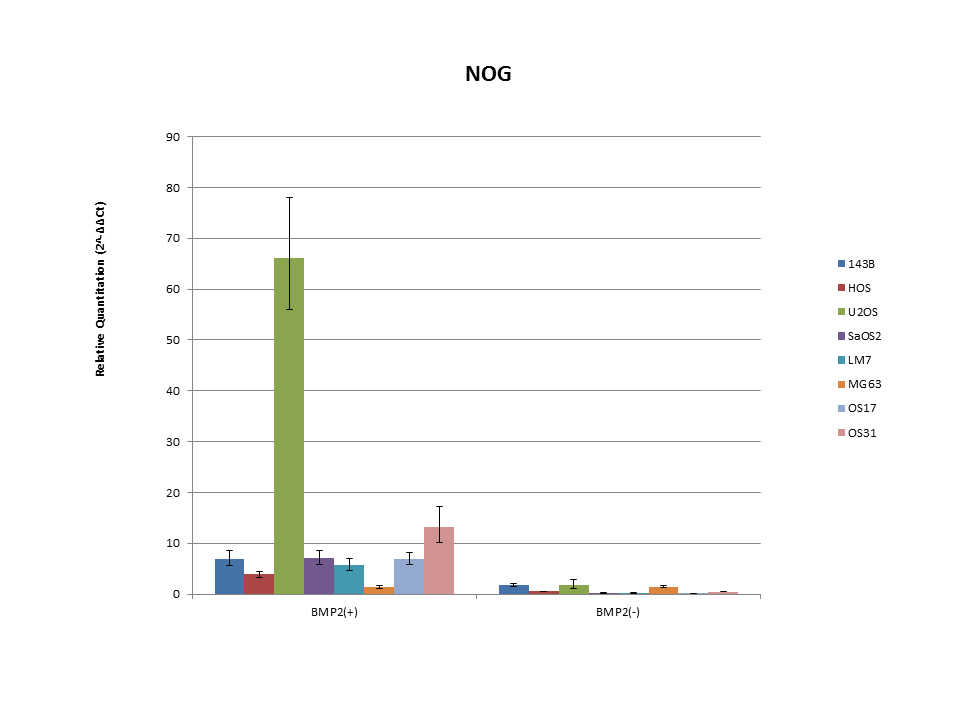

Supplement: S4 Fig — There was a signficant difference in expression between the experimental group exposed to BMP-2 and the control group that was not exposed to BMP-2. (TIF) [file pone.0173322.s004.tif]

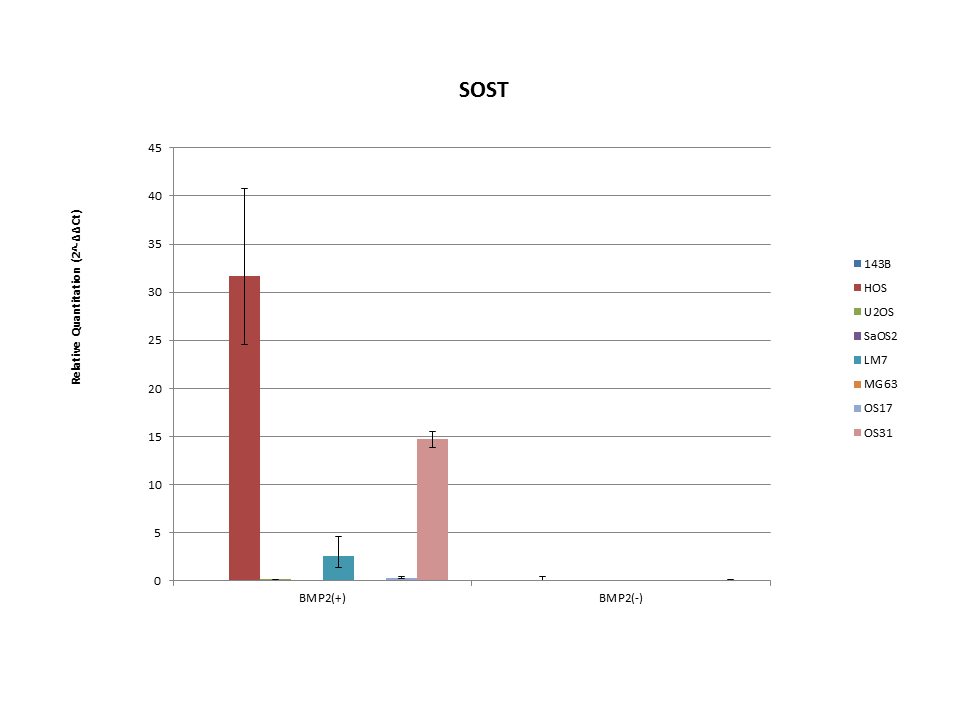

Supplement: S5 Fig — There was a signficant difference in expression between the experimental group exposed to BMP-2 and the control group that was not exposed to BMP-2. (TIF) [file pone.0173322.s005.tif]

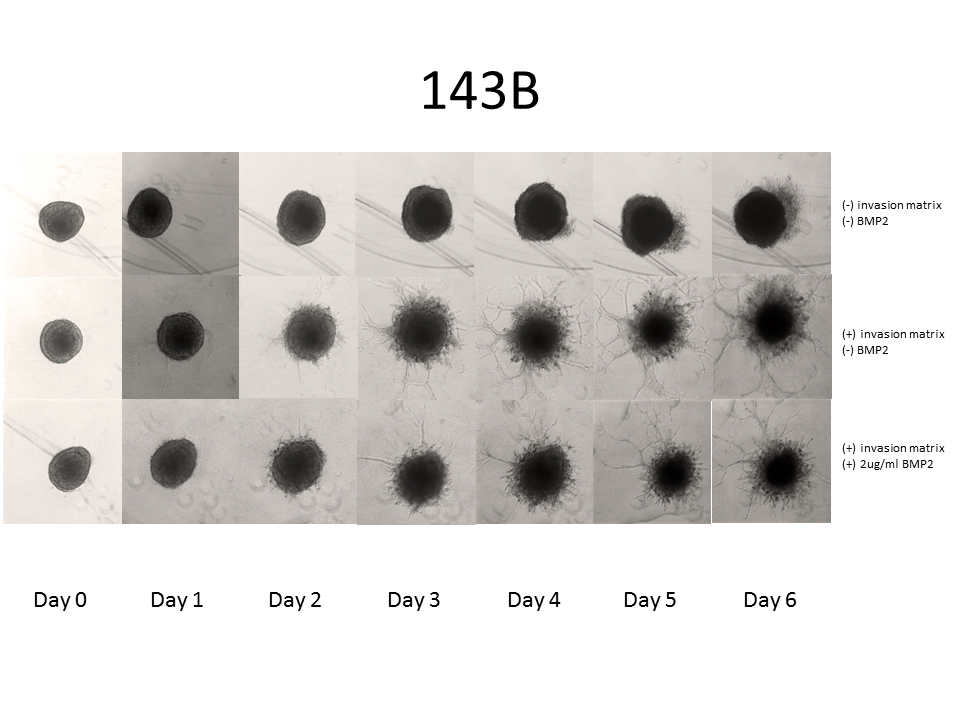

Supplement: S6 Fig — The cell line without BMP-2 and invasion matrix, with invasion matrix and without BMP-2, and with BMP-2 and the invasion were compared and did not demonstrate a qualitative difference. (TIF) [file pone.0173322.s006.TIF]

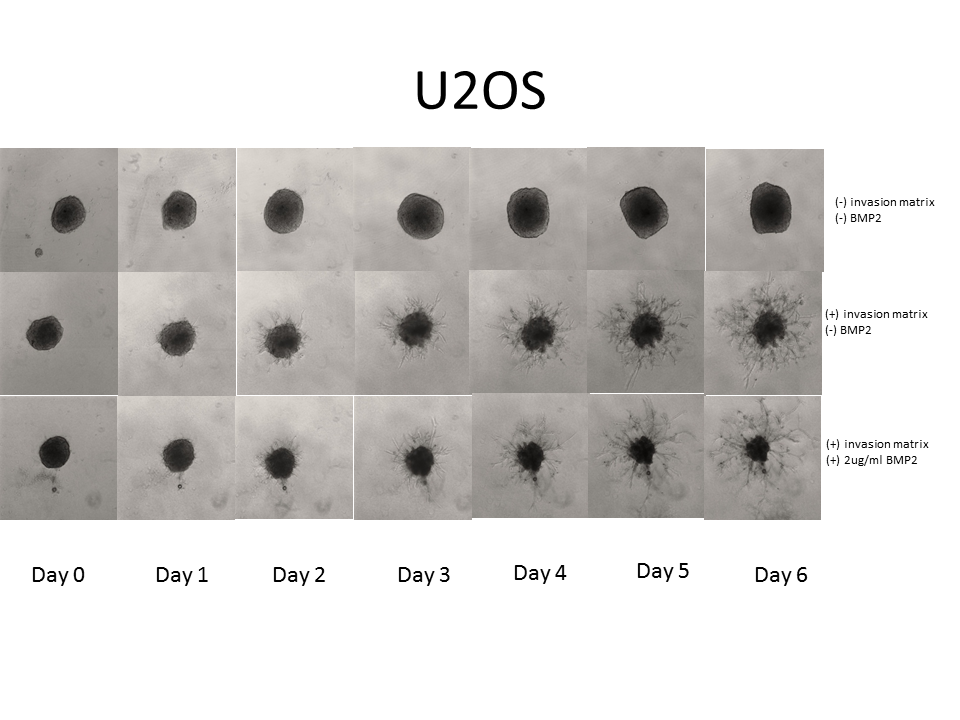

Supplement: S7 Fig — The cell line without BMP-2 and invasion matrix, with invasion matrix and without BMP-2, and with BMP-2 and the invasion were compared and did not demonstrate a qualitative difference. (TIF) [file pone.0173322.s007.tif]

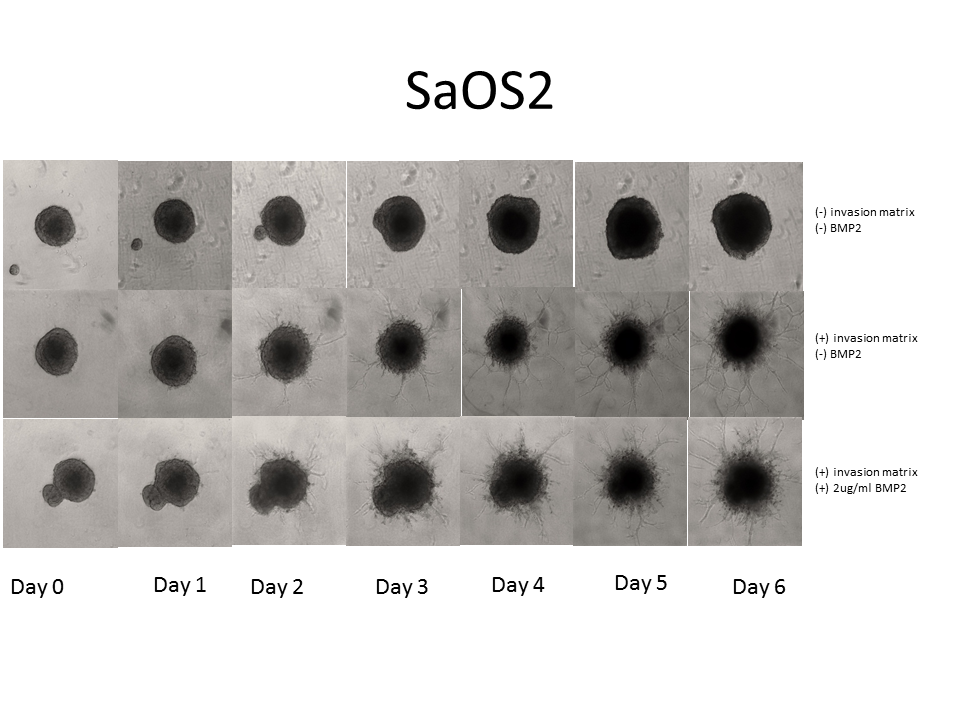

Supplement: S8 Fig — The cell line without BMP-2 and invasion matrix, with invasion matrix and without BMP-2, and with BMP-2 and the invasion were compared and did not demonstrate a qualitative difference. (TIF) [file pone.0173322.s008.tif]

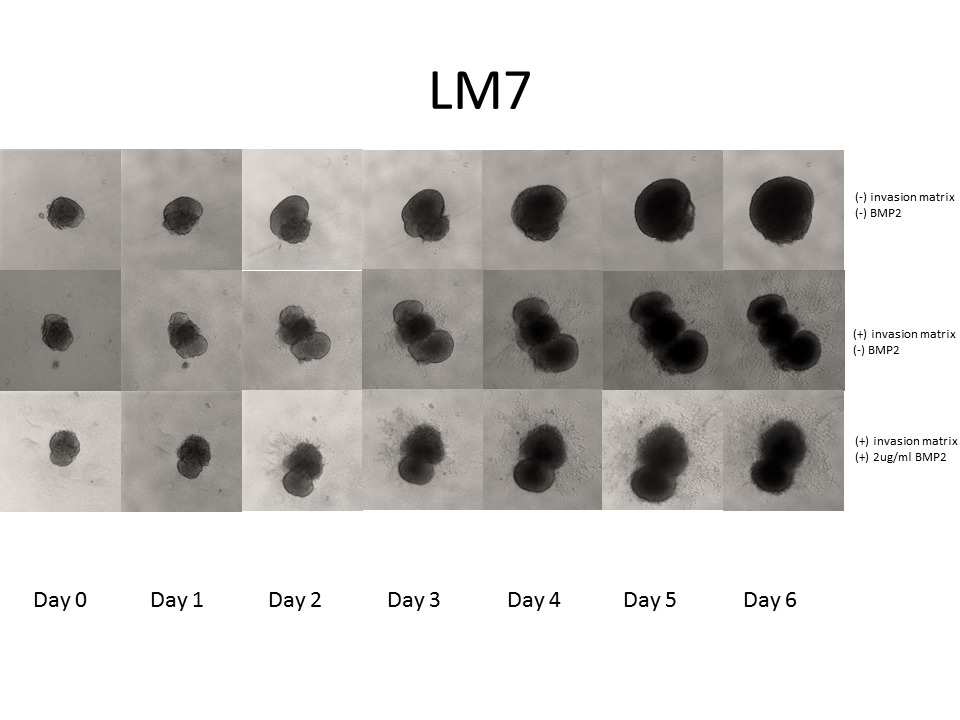

Supplement: S9 Fig — The cell line without BMP-2 and invasion matrix, with invasion matrix and without BMP-2, and with BMP-2 and the invasion were compared and did not demonstrate a qualitative difference. (TIF) [file pone.0173322.s009.tif]

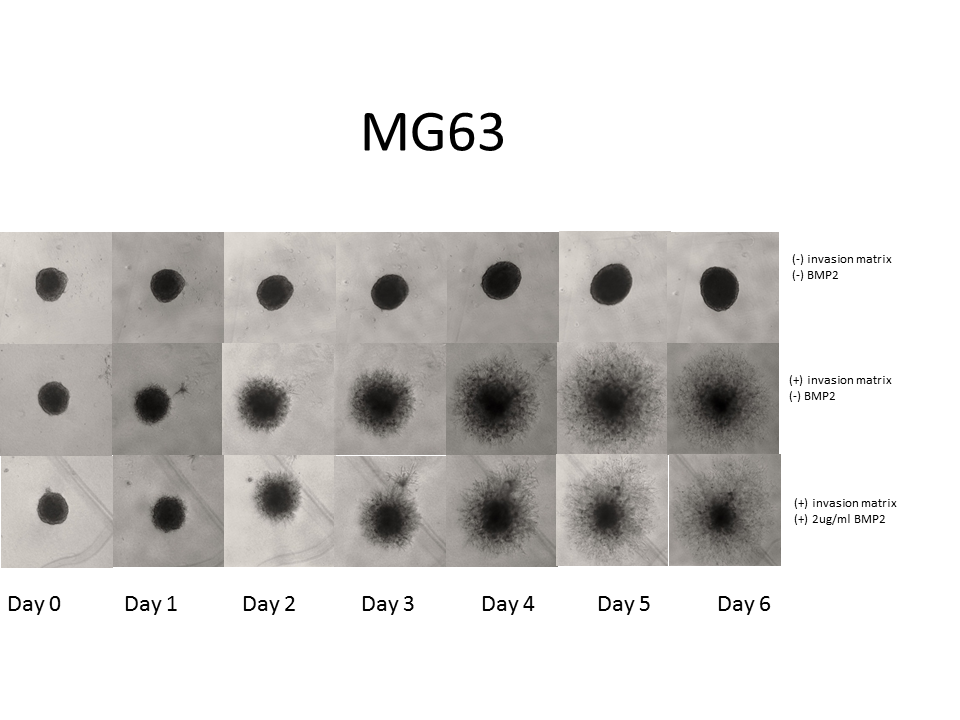

Supplement: S10 Fig — The cell line without BMP-2 and invasion matrix, with invasion matrix and without BMP-2, and with BMP-2 and the invasion were compared and did not demonstrate a qualitative difference. (TIF) [file pone.0173322.s010.tif]

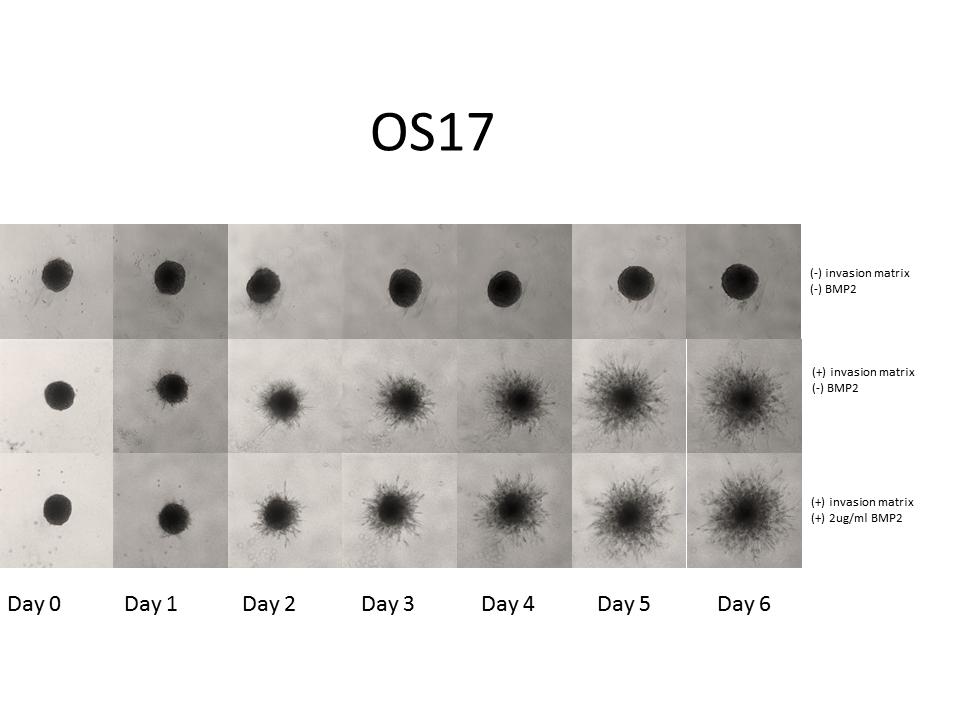

Supplement: S11 Fig — The cell line without BMP-2 and invasion matrix, with invasion matrix and without BMP-2, and with BMP-2 and the invasion were compared and did not demonstrate a qualitative difference. (TIF) [file pone.0173322.s011.tif]

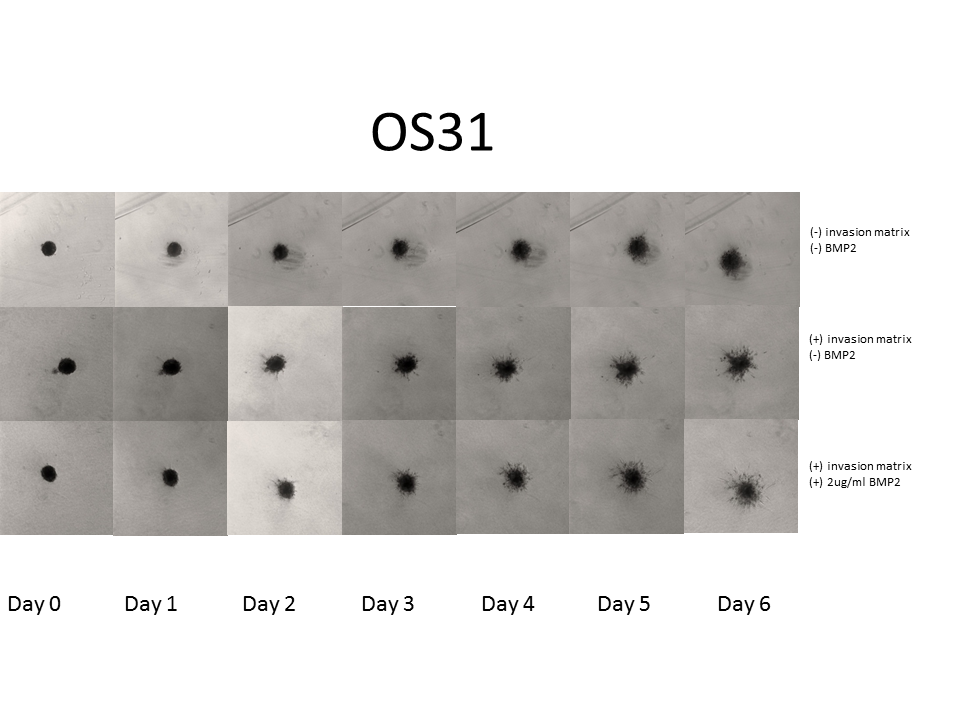

Supplement: S12 Fig — The cell line without BMP-2 and invasion matrix, with invasion matrix and without BMP-2, and with BMP-2 and the invasion were compared and did not demonstrate a qualitative difference. (TIF) [file pone.0173322.s012.TIF]
